# Relationship between air pollution and childhood atopic dermatitis in Chongqing, China: A time-series analysis

**DOI:** 10.3389/fpubh.2022.990464

**Published:** 2022-10-06

**Authors:** Pan Luo, Dan Wang, Jia Luo, Shan Li, Meng-meng Li, Hao Chen, Yong Duan, Jie Fan, Zheng Cheng, Ming-ming Zhao, Xing Liu, Hua Wang, Xiao-yan Luo, Li Zhou

**Affiliations:** ^1^Department of Epidemiology, School of Public Health, Chongqing Medical University, Chongqing, China; ^2^Department of Dermatology, Children's Hospital of Chongqing Medical University, Chongqing, China; ^3^Ministry of Education Key Laboratory of Child Development and Disorders, Chongqing, China; ^4^National Clinical Research Center for Child Health and Disorders, Chongqing, China; ^5^Nan'an District Center for Disease Control and Prevention, Chongqing, China

**Keywords:** air pollution, atopic dermatitis, time-series study, generalized additive model, pediatric outpatient visits

## Abstract

**Background:**

The prevalence of atopic dermatitis (AD) in children has increased substantially in China over past decades. The ongoing rise in the prevalence stresses the important role of the environmental factors in the pathogenesis of AD. However, studies evaluating the effects of air pollution on AD in children are scarce.

**Objective:**

To quantitatively assess the association between air pollution and outpatient visits for AD in children.

**Methods:**

In this time-series study, we collected 214,747 children of AD from January 1, 2015 to December 31, 2019 through the electronic data base in the Children's Hospital of Chongqing Medical University. The number of daily visits was treated as the dependent variable, and generalized additive models with a Poisson like distribution were constructed, controlling for relevant potential confounders and performing subgroup analyses.

**Results:**

Each 10 μg/m^3^ increase in PM_2.5_, PM_10_, SO_2_, NO_2_ and each 1 mg/m^3^ increase in CO concentrations was significantly associated with a 0.7% (95% CI: 0.2, 1.3%), 0.9% (95% CI: 0.5, 1.4%), 11% (95% CI: 7.5, 14.7%), 5.5% (95% CI: 4.3, 6.7%) and 10.1% (95% CI: 2.7, 18.2%) increase of AD outpatient visits on the current day, respectively. The lag effect was found in SO_2_, PM_10_, and NO_2._ The effects were stronger in cool season and age 0–3 group.

**Conclusions:**

Our study suggests that short-term exposure to ambient air pollution contributes to more childhood AD outpatient visits in Chongqing, China.

## Introduction

Atopic dermatitis (AD), also known as atopic eczema, is a common chronic inflammatory skin disorder that has high morbidity in young children (1). It is characterized by a chronic or relapsing disease course of eczematous lesions over dry skin (2). According to the estimates of the International Study of Asthma and Allergies in Childhood (ISAAC), AD affects 15–20% of children and 1–3% of adults globally (3). In China, the prevalence of childhood AD has increased rapidly in the past two decades in parallel with the advancement in industrialization and urbanization, suggesting an environmental effect (4, 5). Compared to adults, children are more susceptible to air pollution, because of their lower breathing zone and shorter airways (6). In the US, 10–30% of AD patients persist in adulthood or even throughout life (7). AD significantly reduces patients' quality of life and increases the burden of families and society (8). Severe childhood AD may cause high incidence of psychosocial disorders and related complications (7) and is the incipient signal of asthma or atopic rhinitis (5). Therefore, the assessment of the effects of environmental factors on AD, especially the role of air pollution in AD, will help to expand our understanding and develop better strategies for the prevention of AD.

AD is caused by a complex interaction of genetic, immunologic, and environmental factors (9). Recent epidemiological studies have shown a relationship between air pollution (such as PM, NO_2_ or O_3_) and AD (10–13). A time-series study has reported significant relationships of O_3_, PM_10_ and SO_2_ levels with medical care visits for AD (14). Another similar study has found ambient PM_10_, SO_2_, NO_2_ and CO all positively associated with AD (15). Unlike asthma and atopic rhinitis, the evidence for the association between AD and air pollution is still scarce and controversial with relatively less research, small samples, limitations of study designs and other issues. Investigations to clarify the role of air pollution in childhood AD remains a challenge (16). With the rapid development of industrialization and urbanization over the past two decades, China has been experiencing the worst ambient air pollution in the world (17). Previous studies on the relationship between air pollution and AD in China were restricted to high-income cities [such as Beijing (13), Guangzhou (12), and Shanghai (18)], with limited information from low- and middle-income cities, despite much higher air pollution levels in these cities.

Chongqing, the largest municipality directly under the central government in China, is a provincial administrative unit. It's a middle-income and major heavy industry city with an area of 5472.68 km^2^ and a population of >21.1 million in main urban area in 2021. However, Chongqing has been experiencing the severe air pollution, and is not conducive to diffuse these pollutants because of its special mountainous urban landform. What's more, the morbidity of AD in children aged 1–7 years in Chongqing was 17.63%, higher than that of in Beijing (9.00%) and Shanghai (10.72%), ranking fourth among the 12 major cities in China (5). Hence, investigation of the role of air pollution contribute to the risk of childhood AD in Chongqing has become increasingly relevant.

In the present study, in order to investigate the effects of air pollution (PM_2.5_, PM_10_, SO_2_, NO_2_, CO, O_3_) on AD, we used generalized additive models (GAMs) to evaluate the impact of air pollutants on daily number of outpatient visits for AD. The object is to identify environmental triggers in individual properly and reduce the occurrence and symptomatic worsening of AD, which can provide a evidence for the effective control of AD in children.

## Methods

### Study area and data collection

We conducted this time-series study in the urban areas in Chongqing and obtained daily outpatient records for AD (children aged 0–18 years) between January 1, 2015 and December 30, 2019 from the electronic medical system of Children's Hospital of Chongqing Medical University. The hospital is located in the center of Chongqing and is also one of the highest-level hospitals for pediatric diseases in China. The record includes general personal information, clinical diagnosis and ICD code. The patients for AD were confirmed by the ICD-10 code as L20.9. Patients who were living out of the urban area were excluded. All participants gave written informed consent, and the study was performed with the approval of the Ethics Committee of Chongqing Medical University in Chongqing, China.

### Environmental data

The 24-h average concentration of PM_2.5_, PM_10_, NO_2_, SO_2_, CO and the maximum 8-h concentration for O_3_ were collected from Chongqing Environmental Protection Bureau. There were 17 fixed monitoring stations for air pollution in Chongqing and evenly distributed in the urban area. The mean values from all monitoring stations were used to express the air pollutant exposure levels. The daily temperature and humidity information in the same period were collected from China Meteorological Data Service Center.

### Statistical analysis

The relationship between air pollution and meteorological variables was analyzed by Spearman correlation. In order to evaluate the acute and lag effects of air pollution on AD. We used a generalized additive model with quasi Poisson links to estimate the association between air pollution exposure and AD outpatient visits. According to previous studies (12, 18), penalized smoothing spline functions were used to control long-term trends (df = 7/year), seasonal patterns, temperature (df = 3), relative humidity (df = 3) and an indicator variable for the day of the week and holiday. The model evaluates each lag effect separately.

We used the excess risk percentage (ER%) and its 95% confidence interval (Cl) to determine hazard degree of air pollution. The calculation formula of ER% is as follows:


(1) $$\begin{array}{l} \text{ER\%}={\left(\text{RR}-1\right)}^{*}\;100\% \end{array}$$

To evaluate the impact cycle of air pollution on AD outpatient visits, we explored the impact of air pollution with different lag structures: single day lag from lag0 to lag5. Three moving average lag structures (lag01, lag03, and lag05) was selected to evaluate the cumulative effect of air pollution on AD.

### Stratified analysis

To explore the impact of air pollution on patients with AD in different seasons, ages and genders. We defined the warm season from May 1 to September 30 and the cool season from October 1 to April 30. The patients were divided into two age groups: lower age group was age 0–3; higher age group was age 4–18. Gender was recorded as male or female. The difference between groups was calculated by *Z*-test, and the formula is as follows:


(2) $$\begin{array}{l} Z=\frac{\beta 1-\beta 2}{\sqrt{Sd_{1}^{2}}-\sqrt{Sd_{1}^{2}}} \end{array}$$

Among β*1* and β*2* is the regression coefficient of each subgroup, and *Sd*_1_ and *Sd*_2_ are the standard error of each subgroup. R version 3.2.3 was used for all statistical analyses, *P* < 0.05 was considered statistically significant, and all *P* values were tested by bilateral test.

### Sensitivity analysis

We examined the robustness of our results by: (a) fitting two-pollutant model. Pollutants with correlation coefficients <0.7 were incorporated into the model to reduce collinearity. (b) changing the degrees of freedom in the S function of time variable (8–10 df) and meteorological variables (4–6 df).

## Results

The daily outpatient number of children with AD, air pollution concentration and meteorological factors are summarized in Table 1. The mean daily concentrations of PM_2.5_, PM_10_, SO_2_, NO_2_, O_3_, and CO were 45.8, 71.1, 11.5, 43.3, 73.3 μg/m^3^, and 1.0 mg/m^3^, respectively. O_3_ was higher in warm season and lower in cold season, other air pollutants were the opposite. During this study period. A total of 214,747 records of AD visits were included. Among these visits 124,374 were male patients (57.9%) and 177,307 were age 0–3 (82.6%). The cases were mainly concentrated in winter and spring, and less in summer and autumn. The mean daily number of AD outpatient visits was 117.7 and there were 3 days with zero cases in the study.

**Table 1:** Daily air pollution, meteorological data, and outpatient visits for AD in Chongqing, China, from January 1, 2015, to December 30, 2019.

	**Mean** ±**SD**			**Min**	**P25**	**P50**	**P75**	**Max**
	**Whole study**	**Cool season**	**Warm season**					
**Air pollutants**								
PM_2.5_ (μg/m^3^)	45.8 ± 26.8	54.4 ± 30.1	34.0 ± 14.7	9.0	28.0	38.0	56.0	212.0
PM_10_ (μg/m^3^)	71.1 ± 35.2	81.2 ± 40.0	57.1 ± 27.0	12.0	47.0	62.0	85.0	293.0
SO_2_ (μg/m^3^)	11.5 ± 5.5	13.0 ± 6.2	9.3 ± 3.2	4.0	8.0	10.0	14.0	42.0
NO_2_ (μg/m^3^)	43.3 ± 11.4	47.3 ± 11.6	37.7 ± 8.5	16.0	35.0	42.0	50.3	96.0
O_3_ (μg/m^3^)	73.3 ± 46.2	48.4 ± 32.0	108.0 ± 40.0	4.0	33.0	65.0	108.0	228.0
CO (mg/m^3^)	1.0 ± 0.2	1.1 ± 0.3	0.8 ± 0.1	0.4	0.8	0.9	1.1	3.4
**Meteorology**								
Temperature (°C)	18.6 ± 7.7	13.4 ± 5.2	25.7 ± 4.3	−1.0	11.0	19.0	24.0	36.0
Relative humidity (%)	74.6 ± 11.6	76.7 ± 10.4	71.7 ± 12.6	28.0	67.0	76.0	83.0	97.0
**Outcome (atopic dermatitis)**								
Daily admission	117.7 ± 54.1	146.3 ± 49.5	78.1 ± 29.7	0	73.0	110.0	158.0	294.0
**Stratified by age**								
< 4 years old	97.2 ± 49.4	124.3 ± 44.5	59.6 ± 25.7	0	56.0	89.0	135.0	271.0
4–18 years old	20.52 ± 10.8	22.0 ± 11.5	18.4 ± 9.5	0	13.0	18.0	26.0	78.0
**Stratified by gender**								
Female	49.5 ± 22.3	60.5 ± 21.0	34.4 ± 13.7	0	32.0	47.0	65.0	122.0
Male	68.2 ± 33.0	85.8 ± 30.2	43.7 ± 17.4	0	42.0	63.0	93.0	172.0

SD, Standard deviation; PM2.5, particulate matter <2.5 μm in aerodynamic diameter; PM10, particulate matter <10 μm in aerodynamic diameter; SO2, sulfur dioxide; NO2, nitrogen dioxide; O3, Ozone.

Cool season: October 1 to April 30; Warm season: May 1 to September 30.

P25, the first quartile; P50, the second quartile (median); P75, the third quartile; Min, minimal value; Max, maximal value.

The Spearman's correlation coefficients between air pollution and meteorological factors are displayed in Table 2. PM_2.5_, PM_10_, SO_2_, NO_2_, and CO were positively correlated. O_3_ was negatively associated with other pollutants. Temperature was positively correlated with O_3_, negatively correlated with other air pollutants. Relative humidity was positively correlated with CO, negatively correlated with PM_10_ and SO_2_.

**Table 2:** Spearman's correlation between daily mean air pollutants concentrations and meteorological factors in Chongqing, China.

	**PM_2.5_**	**PM_10_**	**SO_2_**	**NO_2_**	**O_3_**	**CO**	**TEMP**	**RH**
PM_2.5_	1.00							
PM_10_	0.96^**^	1.00						
SO_2_	0.63^**^	0.69^**^	1.00					
NO_2_	0.74^**^	0.78^**^	0.61^**^	1.00				
O_3_	−0.32^**^	−0.20^**^	−0.15^**^	−0.24^**^	1.00			
CO	0.71^**^	0.67^**^	0.54^**^	0.64^**^	−0.49^**^	1.00		
TEMP	−0.45^**^	−0.35^**^	−0.26^**^	−0.40^**^	0.77^**^	−0.50^**^	1.00	
RH	0.02	−0.10^**^	−0.22^**^	0.02	−0.49^**^	0.18^**^	−0.42^**^	1.00

** At the 0.01 level (two tailed), the correlation was significant.

**Figure 1:**
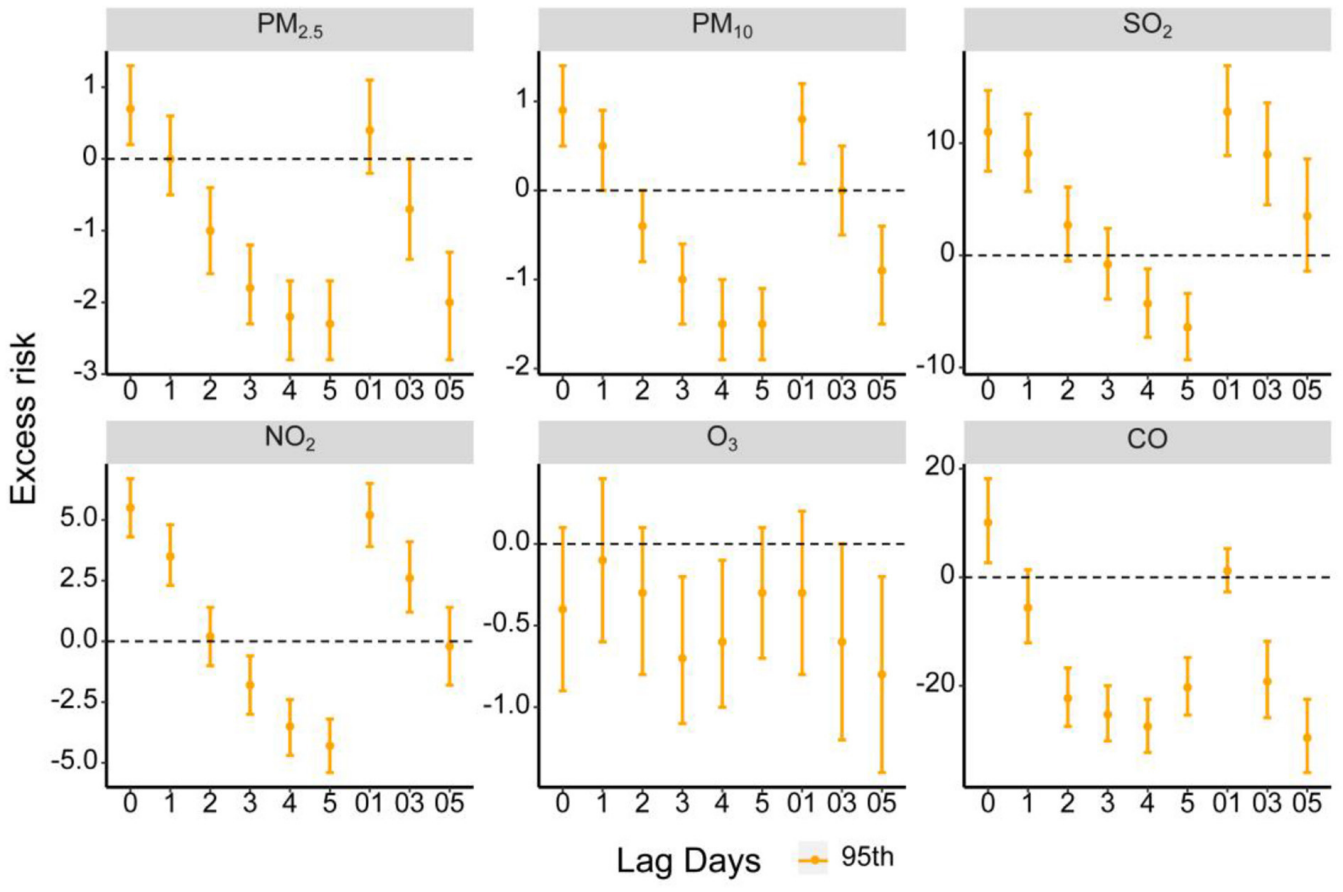
Excess risk (ER, %) and 95% confidence intervals (CIs) for AD of each air pollutant along different lag days in Chongqing, China.

Figure 1 shows that each 10 μg/m^3^ increase in PM_2.5_, PM_10_, SO_2_, NO_2_ and each 1 mg/m^3^ increase in CO concentrations was significantly associated with a 0.7% (95% CI: 0.2, 1.3%), 0.9% (95% CI: 0.5, 1.4%), 11% (95% CI: 7.5, 14.7%), 5.5% (95% CI: 4.3, 6.7%) and 10.1% (95% CI: 2.7, 18.2%) increase of AD outpatient visits on the day of exposure (lag0), respectively. The delayed effects lasted up to 1 day for PM_10_ [0.5% (95% CI: 0, 0.9%)], SO_2_ [9.1% (95% CI: 5.7, 12.6%)] and NO_2_ [3.5% (95% CI: 2.3, 4.8%)]. The cumulative effects appeared at lag01 for PM_10_ [0.8% (95% CI: 0.3, 1.2%)], lag03 for SO_2_ [9.0% (95% CI: 4.5, 13.6%)] and NO_2_ [2.6% (95% CI: 1.2, 4.1%)].

**Figure 2:**
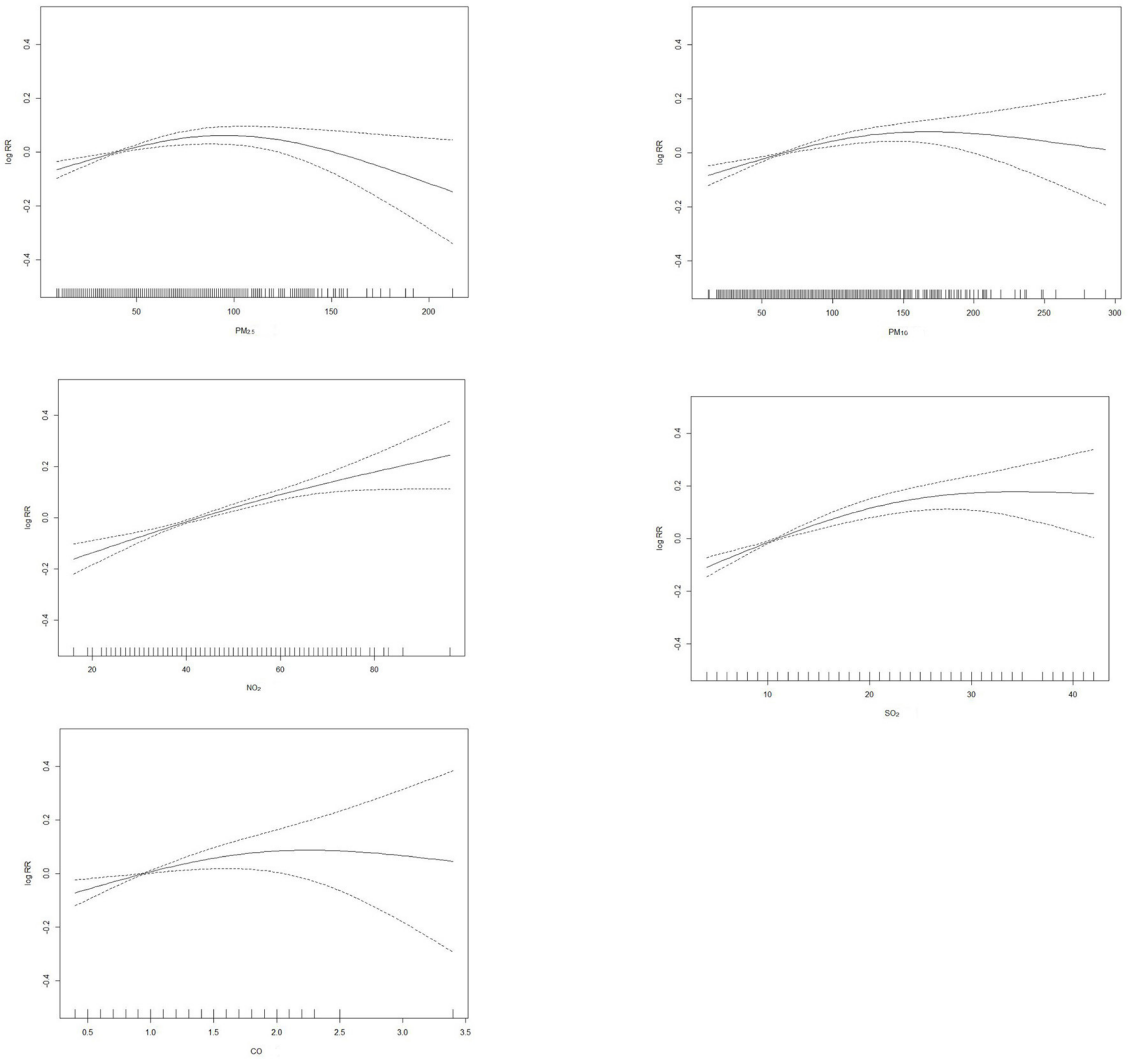
Dose-response curves for different air pollutants in Chongqing, China.

Since contaminants showed the strongest effects on the day of elevated concentrations, we explored the exposure-response relationship of PM_2.5_, PM_10_, SO_2_, NO_2_, and CO with that day's outpatient visit and present it in Figure 2. We observed that the exposure–response relationship for SO_2_ and NO_2_ was almost linear. The exposure–response curves for PM_2.5_, PM_10_, and CO were partial departure from linearity.

Percentage changes with 95% CI in AD outpatient visits stratified by gender, age, and season are listed in Table 3. We did not observe any significant difference effect modification across sex. For the age factor, each pollutant seems to be more harmful to age0-3 group, in which there was a significant statistical difference in SO_2_. For the season factor, NO_2_ is more harmful in the cold season, and the difference effect modification of other pollutants did not reach the statistical threshold.

**Table 3:** Percentage changes with 95% confidence intervals (CIs) in AD outpatient visits stratified by gender, age and season.

	**PM_2.5_**		**PM_10_**		**SO_2_**		**NO_2_**		**CO**	
	**ER (95% Cl)**	***p*** **value**	**ER (95% Cl)**	***p*** **value**	**ER (95% Cl)**	***p*** **value**	**ER (95% Cl)**	***p*** **value**	**ER (95% Cl)**	***p*** **value**
Male	0.7 (0.1–1.3)		0.9 (0.5–1.3)		10.9 (7.2–14.7)		5.5 (4.2–6.8)		9.9 (2.1–18.3)	
Female	0.8 (0.1–1.4)	0.391	1.0 (0.5–1.5)	0.374	11.4 (7.4–15.5)	0.393	5.5 (4.1–6.9)	0.398	10.5 (2.2–19.5)	0.397
Age 0–3	0.9 (0.2–1.5)		1.1 (0.6–1.5)		13.0 (9.1–−17.0)		6.0 (4.7–7.4)		13.0 (4.9–22.0)	
Age 4–18	0.4 (– 0.7–1.4)	0.293	0.4 (– 0.3–1.2)	0.145	2.8 (– 3.3–9.2)	0.012	3.5 (1.3–5.7)	0.065	– 0.8 (– 12.4–12.4)	0.084
Warm	1.2 (– 0.1–2.6)		1.4 (0.5–2.2)		8.3 (2.0–14.9)		2.4 (0.4–4.4)		10.0 (– 3.4–25.3)	
Cold	0.7 (0.1–1.4)	0.301	0.9 (0.4–1.4)	0.256	12.6 (8.1–17.4)	0.227	6.1 (4.4–7.8)	0.009	7.7 (−0.9–17.1)	0.385

Supplementary Table 1 shows the results of the two-pollutant model. Further adjustment for exposure to other pollutants did not statistically change the association between NO_2_ and AD, it suggests NO_2_ has a strong independent effect. Other pollutants may be due to collinearity, so that the estimated excess risk is greatly reduced or even significance disappears after adding another pollutant to the original model. Supplementary Table 2 shows that the associations of air pollution exposures with AD outpatient visits did not differ statistically after changing temperature and humidity freedom.

## Discussion

Our study found that short-term exposure to PM_2.5_, PM_10_, SO_2_, NO_2_, and CO were significantly associated with the increase of AD outpatient visits, indicated that the ambient air pollution posed an acute risk to AD patients. In addition, we found that age and season factors can significantly adjust the relationship between air pollution and AD outpatient visits, but no significant difference was found between different gender groups. The sensitivity analysis further verified the robustness of the overall results.

The findings of PM_2.5_ and PM_10_ of our study were consistent with previous studies. A research in Beijing which revealed that PM_2.5_ per IQR rise (70.8 μg/m^3^) would increase AD outpatient visits by 4.72% (19). Another study investigating the impact of air pollution on AD in Jencheon, Korea found every 10 μg/m^3^ increase in PM_10_ levels has resulted in 1.08% increase in AD outpatient attendance (13). However, our study observed higher excess risks in NO_2_, SO_2_ as compared to related domestic studies (19, 20). The main reason for the difference may be that we chose children as the study sample. Pediatric AD patients may be more sensitive to air pollutants for reasons such as immune immaturity, and therefore higher excess risk was observed (14). In China, the evidence of the effect of O_3_ exposure on AD risk is still controversial. For example, Hu's study in Shanghai, China found a promoting effect of O_3_ on outpatient visits for AD (17). Wang's study in Taiwan, was consistent with ours, found no association between O_3_ and the development of AD (21). At the same time, it is also possible that the unstable and decomposable nature of O_3_ makes it less effective, which in turn leads us not to observe its deleterious effects (22). The research about O_3_ needs to continue in depth, especially in the current situation where low-concentration O_3_ has been applied to the treatment of skin diseases (23). Furthermore, a clear effect of CO appeared in the single pollutant model, but not in the two-pollutant model, suggesting that CO may not be an independent risk factor for AD outpatient visits. P. Kathuria's study in the United States also did not find the relationship of CO with AD (24). While, given the limitation of sample and study area, the link between CO and the occurrence of AD requires further study.

The identification of individuals at potential risk has important public health implications. Several studies did not observe any significant effect modification by gender on the association of air pollution with AD (11, 25), which is consistent with our study. On seasonal factors, we found that the effect of PM_2.5_ on AD was significant in the cold season but not in the warm season, and the effect of NO_2_ was also stronger in the winter. This season-related exacerbation may explained by the decrease of temperature in winter, which leads to the reduction of skin oil and sweat secretion and the decrease of skin barrier function in AD patients. Besides, factors such as UV exposure and vitamin D status may also affects the diseases (26), Therefore, the multiple negative effects in winter of Chongqing resulted in the increased AD outpatient visits.

With respect to age factors, we found that the associations between PM_2.5_, PM_10_, SO_2_, CO, and AD outpatient visits were significant in the age 0–3 group only, not in the children and adolescent group. The effect of SO_2_ was also statistically different between the two age groups. This result suggests that infants are more likely to be affected by air pollution, which leads to higher risk to develop AD. As the outermost layer, acting as the first line of protection from air pollution, skin barrier depends largely on the thickness and lipid content of the epidermis, both of which are directly related to age. The immature skin barrier of infants may facilitates the entry of more air pollutants inciting an inflammatory response. Several studies have shown that pollutant itself can cause skin oxidative stress and disrupt skin barrier integrity by altering transepidermal water loss, inflammatory signaling, stratum corneum pH and the skin microbiome (27, 28). Therefore, future study is needed to clarify the specific mechanisms of pollution induced epidermal barrier dysfunction and to find effective protection methods against the pollutant-driven skin barrier damage.

However, our study also has some limitations. First, we did not collect adult medical record data for comparison, and a subsequent multi center large sample study might be better. Second, we chose mean air pollutant concentrations to represent patient exposure, which is subject to some error in pollution exposure assessment. It may be more precise to select Inverse Distance Weighted (IDW) for evaluation. Third, AD occurrence is also related to genetics and many allergens, but the time-series design does not allow analysis of these factors on an individual level, a time-stratified case-crossover analysis may be used as a follow-up supplement.

## Conclusion

Our study observed an association between short-term exposure to air pollution and daily outpatient visits for childhood AD in Chongqing, China. Noted that the effect of certain air pollutants was stronger in cold season. Our study with a large sample provides new evidence that air pollution will exacerbate the occurrence of AD and that age 0–3 are more vulnerable, further studies should be conducted to help governments and individuals reduce the burden of air pollution on childhood AD.

## Data availability statement

The raw data supporting the conclusions of this article will be made available by the authors, without undue reservation.

## Ethics statement

The studies involving human participants were reviewed and approved by the Ethics Committee of Chongqing Medical University. Written informed consent to participate in this study was provided by the participants' legal guardian/next of kin.

## Author contributions

LZ, PL, and DW carried out the concepts, design, data analysis, and manuscript preparation. JL, SL, M-mL, and HC provided for data acquisition. YD, JF, ZC, M-mZ, and XL collected important background information. HW and X-yL performed manuscript review. All authors have read and approved the content of the manuscript.

## Funding

This study was supported by the funds of the Chongqing Yuzhong District Natural Science Foundation (20210139).

## Conflict of interest

The authors declare that the research was conducted in the absence of any commercial or financial relationships that could be construed as a potential conflict of interest.

## Publisher's note

All claims expressed in this article are solely those of the authors and do not necessarily represent those of their affiliated organizations, or those of the publisher, the editors and the reviewers. Any product that may be evaluated in this article, or claim that may be made by its manufacturer, is not guaranteed or endorsed by the publisher.
