# Supplementary material for: Relationship between air pollution and childhood atopic dermatitis in Chongqing, China: A time-series analysis
Source: Front Public Health. 2022 Oct 6;10:990464. doi: 10.3389/fpubh.2022.990464 (PMC9583006; doi:10.3389/fpubh.2022.990464)
Supplement: Supplementary file 1 [file Data_Sheet_1.docx]

**Table S1.** Excess risk (ER, %) and 95% confidence intervals (CIs) of AD for each pollutant in two-pollutant model

|  |  | ER%(95%Cl) | *P* value |
| --- | --- | --- | --- |
| PM_2.5_ | NULL | 0.7 (0.2-1.3) |  |
|  | +SO2 | -0.7 (-1.4-0.1) | 0.005 |
| PM_10_ | NULL | 0.9 (0.5-1.4) |  |
|  | +SO2 | 0.0 (-0.5-0.6) | 0.019 |
|  | +CO | 0.9 (0.4-1.4) | 0.397 |
| SO_2_ | NULL | 11.0 (7.5-14.7) |  |
|  | +NO2 | 2.3 (-1.9-6.6) | 0.004 |
|  | +PM2.5 | 13.7 (9.1-18.6) | 0.268 |
|  | +PM10 | 10.8 (5.9-15.9) | 0.398 |
|  | +CO | 10.7 (6.9-14.6) | 0.395 |
| NO_2_ | NULL | 5.5 (4.3-6.7) |  |
|  | +SO2 | 5.0 (3.4-6.6) | 0.349 |
|  | +CO | 6.3 (4.8-7.8) | 0.284 |
| CO | NULL | 10.1 (2.7-18.2) |  |
|  | +PM10 | 0.9 (-7.4-10.1) | 0.124 |
|  | +SO2 | 2.1 (-5.3-10.0) | 0.139 |
|  | +NO2 | -8.4 (-15.7--0.5) | 0.002 |

**Table S2.** Excess risk (ER, %) and 95% confidence intervals (CIs) for AD of each air pollutant with different degrees of freedom (df), using lag0.

|  | *df* | PM_2.5_ | PM_10_ | SO_2_ | NO_2_ | CO |
| --- | --- | --- | --- | --- | --- | --- |
| TEMP | 4 | 0.783  (0.206-1.365) | 0.969  (0.554-1.385) | 11.108  (7.593-14.738) | 5.566  (4.325-6.822) | 10.656  (3.245-18.598) |
|  | 5 | 0.783  (0.206-1.365) | 0.969  (0.554-1.385) | 11.108  (7.593-14.738) | 5.547  (4.308-6.800) | 10.656  (3.245-18.598) |
|  | 6 | 0.783  (0.206-1.365) | 0.969  (0.554-1.385) | 11.333  (7.760-15.204) | 5.724  (4.473-6.990) | 10.656  (3.245-18.598) |
| RH | 4 | 0.785  (0.205-1.368) | 0.970  (0.553-1.388) | 11.149  (7.635-14.777) | 5.489  (4.265-6.727) | 10.725  (3.293-18.691) |
|  | 5 | 0.773  (0.191-1.358) | 0.956  (0.539-1.375) | 10.981  (7.467-14.610) | 5.486  (4.249-6.737) | 10.968  (3.510-18.962) |
|  | 6 | 0.832  (0.248-1.419) | 1.005  (0.586-1.425) | 11.255  (7.732-14.894) | 5.575  (4.337-6.828) | 11.695  (4.173-19.760) |
| TIME | 8 | 0.802  (0.223-1.384) | 0.985  (0.570-1.402) | 11.505  (7.977-15.149) | 5.532  (4.305-6.773) | 10.577  (3.181-18.502) |
|  | 9 | 0.826  (0.246-1.409) | 0.993  (0.577-1.410) | 11.604  (8.056-15.268) | 5.651  (4.411-6.904) | 10.494  （3.092-18.427） |
|  | 10 | 0.849  (0.268-1.433) | 1.003  (0.586-1.421) | 11.384  (7.824-15.062) | 5.771  (4.514-7.044) | 11.372  （3.853-19.435） |
